# Changes in the Serum Protein Electrophoresis Profile in Dogs With Pyometra

**DOI:** 10.3389/fvets.2021.626540

**Published:** 2021-03-01

**Authors:** Ji-Seon Yoon, DoHyeon Yu, Jinho Park

**Affiliations:** ^1^College of Veterinary Medicine, Jeonbuk National University, Iksan, South Korea; ^2^College of Veterinary Medicine, Gyeongsang National University, Jinju, South Korea

**Keywords:** serum protein electrophoresis, acute phase protein, biomarker, pyometra, dog

## Abstract

Serum proteins are involved in the regulation of inflammation, and therefore, serum protein profiling provides important insights in diverse inflammatory reactions. Accordingly, concentrations of single APPs, such as the C-reactive protein (CRP), serum amyloid A (SAA), and haptoglobin (Hp), have been described as indicators of inflammatory response in canine pyometra. However, there is little information regarding the overall serum protein profile obtained by SPE in canine pyometra. The present study was thus aimed to identify changes in the serum protein profile to monitor inflammation in dogs with pyometra using serum protein electrophoresis (SPE), in addition to the analysis of the concentration of single acute phase proteins (APPs). By SPE analysis, decreased levels of albumin and elevated levels of α2-globulin and β-globulin were noted in dogs with pyometra. In addition, the concentration of APPs, including the C-reactive protein (CRP), serum amyloid A (SAA), and haptoglobin (Hp), were also elevated in dogs with pyometra. The present study provides fundamental data for inflammatory indicators of canine pyometra.

## Introduction

Pyometra is a common reproductive disorder affecting mostly older female dogs (1) and manifesting as pus accumulation in the uterine lumen. Although the etiology of pyometra likely involves several complex mechanisms, it is generally associated with both hormonal and infectious factors (1). Bacterial uterine infection induces endometrial inflammation, and sometimes leads to sepsis and the systemic inflammatory response syndrome with multiple organ dysfunction, which is life-threatening (1, 2). Therefore, examining possible indicators of diverse inflammatory reactions in canine pyometra may provide important insights into the diagnosis and prognosis of pyometra.

Serum proteins are involved in the stress condition, the regulation of inflammation, and protection against infections which can affect the values of serum protein fractions including acute phase proteins in dogs and in other mammals (2–8). Therefore, serum protein profiling has been used to monitor the severity of inflammatory responses in veterinary medicine (9, 10). Among serum proteins, acute phase proteins (APPs), which are synthesized by hepatocytes upon stimulation by proinflammatory cytokines during an acute phase response, indicate the intensity of inflammation (11, 12). Therefore, concentrations of single APPs, such as the C-reactive protein (CRP), serum amyloid A (SAA) and haptoglobin (Hp) have been described as indicators of inflammatory response in several canine diseases, including pyometra (13, 14).

Alternatively, fractionation techniques have been developed to separate and quantify serum proteins. Serum protein electrophoresis (SPE) is the most widely used laboratory technique for fractionation of serum proteins (9). It separates serum proteins by size and electrical charge, mainly into albumin and α_1_-, α_2_-, β-, and γ-globulins (3, 4). Each fraction contains various proteins functioning as part of the acute phase response and acquired immune responses (9, 10, 15).

In order to obtain accurate results on SPE, appropriate blood sample management is needed. SPE analysis can provide valuable information only if data are accurate and relevant and if their significance is appreciated by the clinicians (16). Careful attention in the process from the collection of the specimens, their transport to the laboratory, sample handling, and analysis are required for obtaining accurate data (17). In addition, previous studies suggest that fresh serum samples or samples refrigerated for no more than 24 h under 20°C should be used (16–18).

In addition to analyzing the concentration of single APPs, overall serum protein profiling by SPE can be a useful diagnostic tool in a wide spectrum of diseases, including infectious and inflammatory diseases. However, there is little information regarding the overall serum protein profile obtained by SPE in canine pyometra. In a previous study, we have reported serum protein profile in SPE and APP concentrations in canine pancreatitis (19). In addition to this, the present study was aimed to investigate serum protein profiles in another inflammatory disease of canine pyometra to gain a better understanding of serum protein concentrations as possible indicators of inflammation.

## Methods

### Dogs

Seven client owned female dogs with diagnosis of opened pyometra were included. Blood serum were obtained from dogs with pyometra who showed anorexia, an enlarged fluid-filled uterus on ultrasonography, neutrophilic leukocytosis and vaginal discharge. All were subsequently treated by ovariohysterectomy. In addition, as control dogs, blood serum were obtained from nine healthy client owned bitches who had regular medical examination without any abnormal clinical conditions and hematologic findings. All experimental procedures were carried out in accordance with the ethical guidelines of the Jeonbuk National University. As only blood serum were obtained from dogs, the anesthesia and euthanasia were not performed in this study.

### Analysis of Serum Protein Profiles by SPE

At the initial presentation, blood samples (3 mL) were collected by direct jugular venipuncture, placed in a tube (BD bioscience, CA, USA) and centrifuged at 1,300 g for 10 min to separate serum. The total serum protein level was measured using a chemistry autoanalyzer (FUJI DRI-CHEM 7000i; Fujifilm Corp, Tokyo, Japan) which is a quantitative immunoassay system for measurement of serum protein and immediately frozen at −80°C. SPE was conducted using agarose gel (Hydrasys2, SEBIA, France) with a protein electrophoresis reagent kit (Hydragel protein(e) 15/30, SEBIA, France), as previously reported (19). The percentage of fractions in SPE was then multiplied by the total protein concentration to quantify the values for each fraction.

### Measurement of Single Concentration of APP

In order to investigate the concentration of single APPs, commercial colorimetric kits were used. Serum CRP was measured using canine CRP immunoassay kit (Tridelta Development, Kildare, Ireland) and SAA concentrations were measured using an immunoassay kit (Tridelta Development, Kildare, Ireland) which has been previously validated for dog (14). In addition, and Hp concentration was determined using kits to evaluate the peroxidase activity of the Hp-hemoglobin complex kit (Tridelta Development, Kildare, Ireland) previously validated for dogs (20). APPs concentrations were analyzed according to the manufacturer's instructions. as previously reported (19).

### Statistical Analysis

Normality test were performed Kolmogorov-Smirnov test of normality, before comparing serum protein profiles of SPE and APP concentrations between dogs with pyometra with those of control dogs. Serum protein profiles in SPE were then compared between dogs with pyometra and control dogs with the unpaired Student's *t*-test. CRP and Hp concentration in dogs with pyometra and control dogs were compared using the unpaired Student's *t*-test. SAA concentration was analyzed by Mann-Whitney test. A *P*-value < 0.05 was considered statistically significant.

## Results

**Figure 1:**
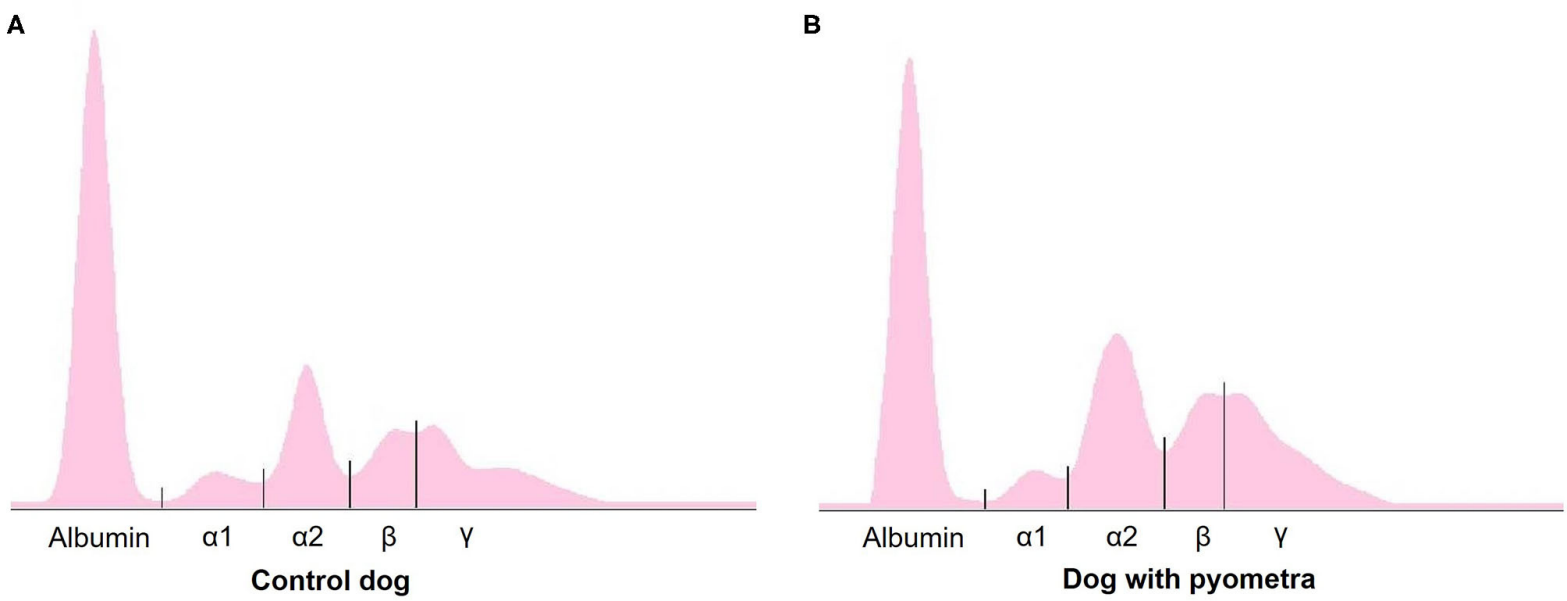
A representative agar gel electrophoretogram in control dogs **(A)** and dogs with pyometra **(B)**.

**Figure 2:**
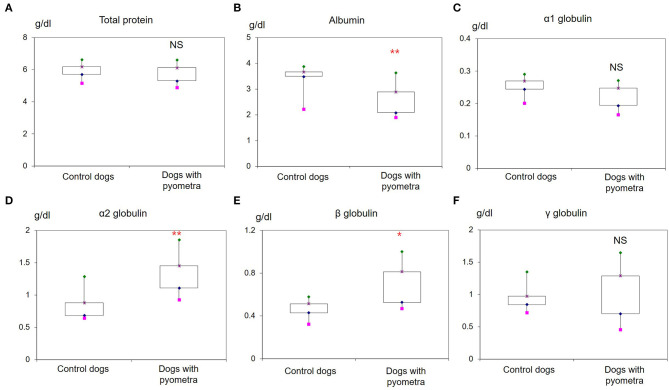
Increased levels of serum proteins in dogs with pyometra separated by serum protein electrophoresis. Significantly reduced levels of albumin **(B)** and elevated levels of α_2_-globulin **(D)** and β-globulin **(E)** were observed in dogs with pyometra compared to the control dogs. Meanwhile, no significant difference was found in the levels of total protein **(A)**, α_1_-globulin **(C)** and γ-globulin **(F)**. NS: no significant difference; *P < 0.05; **P < 0.01.

**Figure 3:**
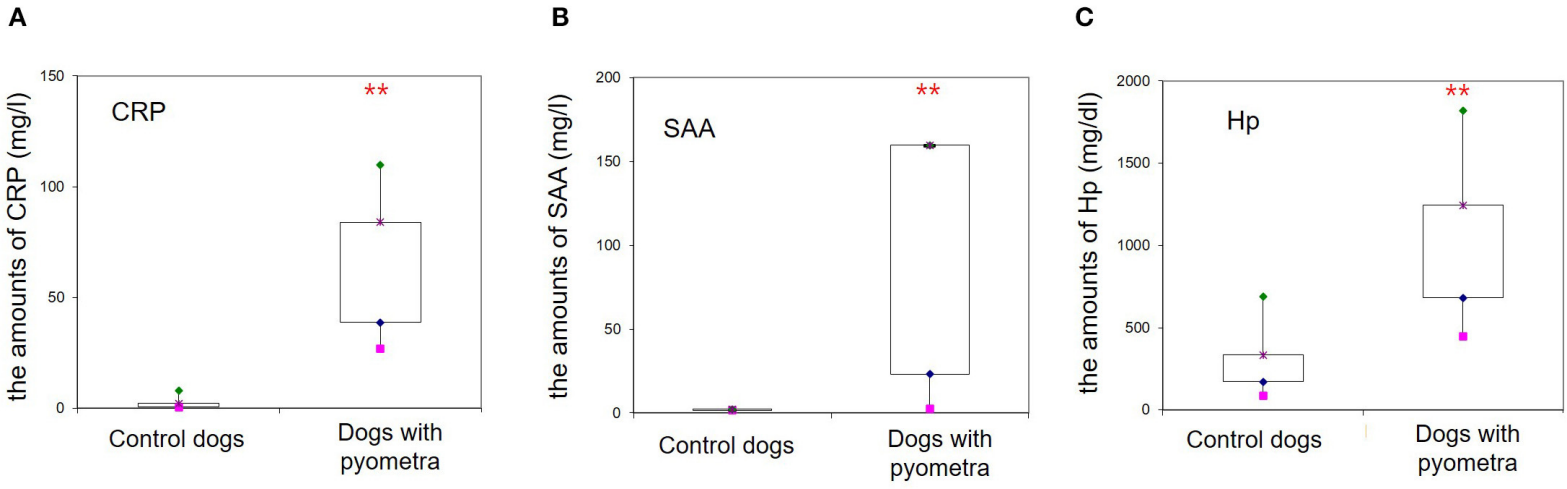
Increased concentrations of acute phase proteins in dogs with pyometra. Significantly elevated levels of the C-reactive protein **(A)**, serum amyloid A **(B)** and haptoglobin **(C)** were observed in dogs with pyometra compared to the control dogs (**P < 0.01).

The mean age of control dogs was 6.8 (range 4–10) years and control dogs belonged to four different breeds: Beagle (*n* = 4), Maltese (*n* = 2), Yorkshire Terriers (*n* = 2) and Shi-Tzu (*n* = 1). The mean age of dogs with pyometra were 10.1 (range 6–15) years and dogs with pyometra belonged to four different breeds: Maltese (*n* = 2), Shi-Tzu (*n* = 2), mongrels (*n* = 2) and Yorkshire Terrier (*n* = 1). Using SPE, serum proteins in both dogs with pyometra and control dogs were separated as five fractions of albumin and α_1_-, α_2_-, β-, and γ-globulins (Figure 1). The quantity of each fraction was calculated as the percentage of the fraction in SPE multiplied by the total protein level measured using the chemistry analyzer. Serum protein levels obtained by SPE in control dogs and dogs with pyometra are shown in Table 1. Among fractions in SPE, significantly reduced albumin and elevated α_2_- and β-globulins were found in dogs with pyometra compared to the control dogs (Figure 2). In addition, the concentration of APPs in control dogs and dogs with pyometra is shown in Table 2. Significant elevation of the concentrations of CRP, SAA, and Hp were observed in dogs with pyometra (Figure 3).

**Table 1:** Serum protein levels in control dogs and dogs with pyometra separated by serum protein electrophoresis (mean ± standard deviation).

	**Control dogs** **(*n* = 9)**	**Dogs with pyometra** **(*n* = 7)**
Albumin (g/dl)	3.46 ± 0.50	2.54 ± 0.65
α1-globulin (g/dl)	0.25 ± 0.03	0.22 ± 0.04
α2-globulin (g/dl)	0.86 ± 0.21	1.30 ± 0.31
β-globulins (g/dl)	0.47 ± 0.08	0.68 ± 0.23
γ-globulin (g/dl)	0.92 ± 0.20	1.01 ± 0.46
Total protein (g/dl)	5.96 ± 0.45	5.76 ± 0.62

**Table 2:** The concentration of acute phase proteins in control dogs and dogs with pyometra (mean ± standard deviation).

	**Control dogs** **(*n* = 9)**	**Dogs with pyometra** **(*n* = 7)**
C-reactive protein (mg/l)	2.43 ± 2.66	62.11 ± 33.67
serum amyloid A (mg/l)	1.82 ± 0.31	98.35 ± 77.68
Total protein (mg/dl)	301.60 ± 194.52	985.38 ± 515.77

## Discussion

In the present study, reduced levels of albumin, elevated levels of α_2_ globulins β-globulin and increased APP concentrations were noted in canine pyometra. Reduced albumin and elevated α2-globulin are characteristic features of acute phase response, as described in our previous study on canine acute pancreatitis (19). On the other hands, elevated β-globulin levels in canine pyometra might be related to concurrent infections. Inflammatory diseases concurrent with infections might lead to elevated α- and β-globulins as a result of elevated serum proteins, such as β_2_-microglobulin, which shows potent antibacterial activity (10). In addition, a previous study on canine pyometra reported elevated α_2_-, β_1_-, β_2_-, and γ-globulins, suggesting elevation of both immunoglobulins and APPs (21). Therefore, there is a possibility that other serum proteins, such as β_2_-microglobulins and immunoglobulins, might affect elevations of β-globulin in dogs with pyometra. Future studies to confirm the concentration of a single protein, such as β_2_-microglobulin and immunoglobulin, will provide further understanding of the elevation of β-globulin in dogs with pyometra.

Elevated APPs, including CRP, SAA, and Hp, were also detected in the present study, in accordance with previous studies (13, 14, 22). CRP and SAA, which are major APPs released rapidly from the liver during the early stage of an acute phase response, increase markedly in canine pyometra (13, 14). In particular, a recent study reported that SAA was significantly higher in septic dogs with pyometra compared to non-septic dogs and could be a useful marker of sepsis (22). In addition, Hp is associated with the expressions of interleukin-6, interleukin-1, and tumor necrosis factor. Elevated Hp in pyometra has also been reported, suggesting Hp as a good candidate for monitoring inflammation in canine pyometra (2, 13). Consistent with previous studies, the present study also revealed elevated APPs, and therefore, CRP, SAA, and Hp might be useful inflammatory indicators of canine pyometra.

A potential limitation of our study was that serum proteins were only collected at initial presentation. The real onset of the inflammatory phenomenon was thus difficult to determine. Therefore, the dogs with pyometra enrolled in the present study might have been at a different stage of the inflammatory process and this could explain the great variability observed among their serum protein levels and APPs concentrations.

Although the use of single APP concentrations to monitor the prognosis of canine pyometra has been described previously (14, 22), there is little information regarding changes in the serum protein profile obtained through SPE during the course of canine pyometra. In dogs with pyometra, not only the acute phase response but also the complement system and immune responses can affect the disease prognosis (2). While APPs are mainly α_2_- and β-globulins in SPE, complements and immunoglobulins are mainly present in β-and γ-globulin fractions (10). Therefore, overall serum protein profiles, including APPs, complements, and immunoglobulins, might provide further information regarding inflammatory response in canine pyometra. Changes in the serum protein electrophoresis profile in dogs with different disease states and after treatment need to be further investigated for a better understanding of the serum protein electrophoresis profile as a prognostic marker of canine pyometra.

In conclusion, changes in the serum protein electrophoresis profile were noted in dogs with pyometra, described as reduced albumin and elevated α_2_- and β-globulin. In addition, elevated CRP, SAA, and Hp were also observed. The present study provides fundamental data for overall serum protein profiles as inflammatory indicators of canine pyometra.

## Data Availability Statement

The raw data supporting the conclusions of this article will be made available by the authors, without undue reservation.

## Ethics Statement

As only blood serum were obtained from dogs, this study did not require institutional ethical approval according to the laws of Bioethics and Safety Act in South Korea. All experimental procedures were carried out in accordance with the ethical guidelines of the Jeonbuk National University. Written informed consent was obtained from the owners for the participation of their animals in this study.

## Author Contributions

J-SY designed the experiments, analyzed the data, and wrote the paper. DY designed and conducted the experiments. JP designed the experiments and wrote the paper. All authors have read and approved the final version of the manuscript.

## Conflict of Interest

The authors declare that the research was conducted in the absence of any commercial or financial relationships that could be construed as a potential conflict of interest.
